# Unraveling the Genome Diversity of *Leishmania* Parasites Using Next-Generation DNA Sequencing Strategies

**DOI:** 10.3390/life15101590

**Published:** 2025-10-11

**Authors:** Alejandro Llanes, Carlos M. Restrepo, Ricardo Lleonart

**Affiliations:** 1 División de Salud Humana y Enfermedades, Instituto de Investigaciones Científicas y Servicios de Alta Tecnología (INDICASAT-AIP), Panama 0801, Panama; allanes@indicasat.org.pa (A.L.); crestrepo@indicasat.org.pa (C.M.R.); 2 Sistema Nacional de Investigación (SNI), Panama 0801, Panama

**Keywords:** *Leishmania*, genome diversity, whole-genome sequencing, next-generation sequencing technologies, population structure, mode of reproduction

## Abstract

Parasites of the *Leishmania* genus are globally distributed and cause various clinical presentations in animals and humans, collectively known as leishmaniasis. The genomes of *Leishmania* and other trypanosomatids exhibit remarkable plasticity, shaped by several distinctive genetic features. Although these features can hinder the application of next-generation DNA sequencing (NGS) technologies, NGS data have been successfully used to characterize the whole-genome diversity of circulating *Leishmania* strains. The results complement and are broadly aligned with previous findings obtained with more traditional methods, offering greater resolution when working with geographically closer strains. In this review, we summarize advances over the past two decades in characterizing the genome diversity of *Leishmania* parasites using NGS strategies. We also discuss the application of these strategies to elucidate other aspects relevant to the epidemiology of these parasites, including their population structure and mode of reproduction. The vast majority of the studies to date have focused on species within the *L. donovani/infantum* complex or the *L.* (*Viannia*) subgenus, highlighting the need to incorporate other relevant underrepresented species and regions from both the Old and New World.

## 1. Introduction

Protozoan parasites of the *Leishmania* genus are the etiological agents of leishmaniasis, a collective name for several clinical presentations affecting animals and humans. These clinical presentations are distinctively caused by more than 20 pathogenic *Leishmania* species, mainly present in tropical and subtropical regions of the world. These pathogenic species are broadly divided into two subgenera termed *L.* (*Leishmania*) and *L.* (*Viannia*), both of which are subdivided into several species complexes. The main pathogenic species causing cutaneous leishmaniasis (CL) in the Old World are those from the *L. major* and *L. tropica* species complexes, including *L. major*, *L. tropica* and *L. aethiopica*. Conversely, species from the *L. donovani*/*infantum* complex, cause the more severe and potentially fatal visceral leishmaniasis (VL). In the New World, CL can be caused by species from the *L. mexicana*/*amazonensis* complex, which also belong to the *L.* (*Leishmania*) subgenus. The epidemiology of leishmaniasis in the New World is further shaped by the presence of the distinct *L.* (*Viannia*) subgenus. *L.* (*Viannia*) parasites are exclusive to Central and South America and comprise two main species complexes: *L. braziliensis*/*peruviana* and *L. panamensis*/*guyanensis*. These species cause mainly CL, but in some cases the parasites can migrate to mucosal tissues of the nasopharyngeal area and cause the more severe and highly disfiguring mucocutaneous presentation (MCL). Recently, the subgenus *L.* (*Mundinia*) was established [1,2], comprising species such as *L. enriettii*, *L. martiniquensis* and *L. orientalis*, some of which are capable of infecting mammals and can cause human disease.

Central to the adaptability of *Leishmania* parasites is their high genomic plasticity, associated with their atypical regulation of gene expression. Unlike many eukaryotes, *Leishmania* and other trypanosomatid parasites lack transcriptional initiation-based regulation of gene expression, relying mostly on post-transcriptional mechanisms involving RNA processing and degradation (see Martinez-Calvillo et al. [3] for a comprehensive review). These constraints in the regulation of gene expression are in part compensated by a remarkable ability to dynamically adjust gene dosage via different forms of genetic amplification [4]. Such mechanisms include the tandem duplication of individual genes leading to copy number variations (CNVs), the amplification of segments of chromosomes in the form of episomic elements, as well as the variations in the somy of entire chromosomes. These changes in the somy of specific chromosomes in individual cells within a rather clonal population, a phenomenon termed mosaic aneuploidy, plays a pivotal role in the parasite’s adaptability, enabling rapid phenotypic shifts in response to environmental stress, including drug pressure [5,6,7]. This intrinsic heterogeneity, often stabilized but reversible when selective pressures subside, allows the population to compensate for deleterious mutations and resist selection bottlenecks [8]. Recently, epistatic interactions between gene CNVs and compensatory transcriptomic responses have also been reported for *Leishmania*, adding another layer of complexity to the regulation of gene expression in this parasite [9].

Despite the technical complexities associated with these distinctive genetic features of *Leishmania* parasites, DNA sequencing strategies have been successfully used to sequence a plethora of *Leishmania* genomes since the *L. major* genome was first released in 2005 [10]. The ability to sequence and compare multiple *Leishmania* genomes has been crucial for understanding complex aspects of the parasite’s biology and epidemiology, as well as the pathophysiology of the different clinical presentations of leishmaniasis. For instance, whole-genome sequencing (WGS) has been used to clarify the possible origin of *L.* (*Viannia*) parasites in South America [11] and to support the hypothesis of the introduction of *L. infantum* in the Americas [12]. Genomic data have also been extensively used to characterize genetic features potentially associated with drug resistance, virulence and disease tropism in different *Leishmania* species [13,14,15,16,17,18]. Comparison of multiple genomes has also been critical to understand the nature and impact of genetic amplification in *Leishmania*, in studies comparing multiple isolates and strains from several species [4,6]. Recent studies have also incorporated the novel single-cell sequencing approach to demonstrate how new karyotypic variants may emerge from near-euploid progenitors, highlighting the dynamic evolution of mosaic aneuploidy within populations [19].

In addition to all these relevant applications, one of the most important contributions of WGS data to the epidemiology of leishmaniasis is the ability to characterize the genetic diversity of circulating *Leishmania* strains in endemic regions. Genomic data has enabled researchers to understand the genetic variability of circulating parasite strains at an unprecedented resolution. By capturing information from entire genomes rather than a limited set of markers, this approach outperforms those based on traditional markers such as multilocus enzyme electrophoresis (MLEE), multilocus microsatellite typing (MLMT) or multilocus sequence typing (MLST). Sequence information from entire genomes, in the form of single-nucleotide polymorphisms (SNPs) or CNVs, provides thousands of markers that greatly enhance the resolution and robustness of phylogenetic and phylogeographic analyses. These data also allow for a more precise characterization of the population structure, hybridization, and the mode of reproduction of circulating parasites. Here, we review the studies that have used WGS data to explore the genome diversity of *Leishmania* since the landmark publications of the first *Leishmania* genomes in the early 2000s. Since this review is focused on genome diversity, we mainly review studies sequencing the genomes of multiple isolates of clinically relevant *Leishmania* species, rather than one or a few representative specimens. We also summarize the evidence in the literature associated with the application of WGS strategies to understand multiple aspects of *Leishmania* biology and epidemiology, including the challenges of applying these approaches in the context of a parasite with markedly distinctive genetic features.

## 2. Onset and Progress of *Leishmania* Genomics: From Sanger to NGS

The first *Leishmania* genome to be completely sequenced (*L. major* strain Friedlin) was part of a hallmark series of articles published in the Nature magazine in 2005, aiming at a comprehensive characterization of trypanosomatid genomes [20]. In this set of articles, the trypanosomatids were represented by the so-called TriTryps: *Trypanosoma brucei* [21], *T. cruzi* [22] and *L. major* [10]. These works were followed by the release in 2007 of the first genome drafts for *L. infantum* (strain JPCM5) and *L. braziliensis* (strain M2904) [23], the latter being the first genome available for the *L.* (*Viannia*) subgenus. All these genomes were sequenced using conventional Sanger sequencing, either on a chromosome-to-chromosome basis or using the whole-genome shotgun strategy, in genome sequencing projects that started years earlier in the late 1990s.

With the advent of the first NGS platforms around 2005, subsequently released *Leishmania* genomes were mainly sequenced using the Roche’s 454 and Illumina platforms, usually in a combined strategy in which the main genome assemblies were generated with 454 mate-paired reads and Illumina reads were then used for error correction and assembly validation. This approach was used to generate the first reference genomes for at least two additional *L.* (*Leishmania*) species: *L. donovani* (strain BPK282A1) [13] and *L. amazonensis* (strain M2269) [24], as well as three *L.* (*Viannia*) species: *L. panamensis* (strain PSC-1) [25], *L. guyanensis* (strain LgCL085) [26] and *L. naiffi* (strain LnCL223) [26]. Similar approaches were also used to generate the first reference genomes for two lizard-infecting *Leishmania* species: *L. tarentolae* (strain Parrot-TarII) [27] and *L. adleri* (strain HO174) [28]. These species belong to an additional *L.* (*Sauroleishmania*) subgenus and are known to mainly infect lizards, although the latter *L. adleri* strain was isolated from an asymptomatic mammalian host. More recently, the Illumina platform was also used to sequence the genomes of three representative species of the *L.* (*Mundinia*) subgenus, namely, *L. enrietti*, *L. martiniquensis* and *L. macropodum* [29].

The first NGS platforms offered several advantages over traditional Sanger sequencing, including a higher throughput and scalability, which markedly reduced sequencing time and cost. However, these platforms posed two main challenges, namely, a higher rate of sequencing errors and a shorter read length, the latter potentially leading to more fragmented genome assemblies. Due to these limitations, the combined use of Roche’s 454 and Illumina required the use of bioinformatic tools to correct errors deriving from one platform with data from the other platform (iCORN [30] or REAPR [31]), close gaps in draft assemblies using mate-paired read information (IMAGE [32]) or contiguate fragmented assemblies guided by a reference genome (ABACAS [33]).

The efficiency, cost-effectiveness and unprecedented read coverage of the Illumina platform also enabled the incorporation of multiple *Leishmania* strains or isolates in genomic studies. In a landmark study, Rogers et al. [4], examined eight *Leishmania* strains and reported a notable genome-wide genetic heterogeneity among species, resulting in the amplification of specific genes and chromosomal regions, as well as the increase in somy of certain chromosomes. This study also provided the first reference genome for *L. mexicana* (strain U1103), although this genome was sequenced using a whole-genome shotgun strategy based on Sanger sequencing. The same year, another study conducted by Downing et al. [13] not only provided the first reference genome for *L. donovani*, but also examined the genome-wide genetic diversity among 16 clinical isolates from the same geographical region. The use of WGS data in this study revealed extensive chromosome and sequence variations, as well as a genetic structure not previously observed by using more traditional methods such as MLST. These pioneer studies pave the way for future works aimed at characterizing the genome diversity of the parasite in different regions of the world, which will be discussed further ahead in this review.

Third-generation DNA sequencing platforms, such as PacBio and Oxford Nanopore Technologies (ONT), have addressed some of the limitations of the first NGS platforms, focusing mainly on sequence read length. These platforms are now capable of generating long reads ranging from tens to hundreds of kilobases, largely mitigating the problem of assembly fragmentation. These platforms enable the production of high-quality, chromosome-level de novo genome assemblies using only long-read data, although Illumina reads are sometimes incorporated to polish these assemblies with programs such as Pilon [34]. Several studies have used this strategy to generate improved versions of *Leishmania* reference genomes, previously assembled with older platforms [35,36,37].

## 3. The Impact of Unique Features of *Leishmania* Genomes on the Application of NGS Strategies

**Figure 1:**
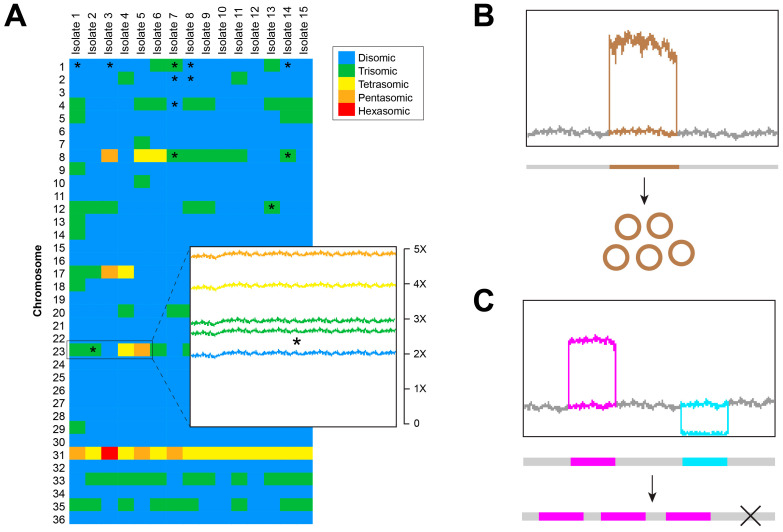
**Schematic representation of the impact of distinctive genetic features of *Leishmania* parasites on NGS data analysis and interpretations.** The figure shows how different genetic changes frequently occurring in *Leishmania* may affect read-depth coverage when aligning sequence reads to a reference genome. (**A**) Variation in the somy of individual chromosomes, usually represented using heatmaps with a color scale indicating somy, can lead to a relatively uniform increase in read-depth coverage along such chromosomes. Chromosomes with fractional somy values (see main text) are often indicated with asterisks (‘*’) or intermediate color shades. (**B**) Episomic amplifications can lead to a local increase in the read-depth coverage, limited to the chromosomal region that is amplified. This increase is usually proportional to the number of copies of the episome. (**C**) Tandem amplification of protein-coding genes, originating gene arrays, can also lead to an increase in read-depth coverage, proportional to the number of copies of the amplified gene. Conversely, total or partial deletion of protein-coding genes (indicated by two black crossing lines) leads to a decrease in read-coverage. The figure shows representative examples of scenarios often observed in practice, not specific results taken from any particular study.

Several distinct features of *Leishmania* genomes can influence both the application and interpretation of NGS strategies when characterizing genomic diversity. One such feature is mosaic aneuploidy per se, which implies that different chromosomes from a given sample can have different somies (Figure 1A). Moreover, since variations in somy occur independently at the level of individual parasites within the population, the estimated somy for certain chromosomes may appear as fractional values or even as values lower than one [4,38]. This poses challenges for most methodologies used to identify genomic variants such as SNPs and CNVs, as well as for downstream analyses that use this information to infer phylogenetic relationships and population structure. Such methods are generally designed for diploid organisms or those with a uniform chromosomal somy in their karyotype, with aneuploidy being the exception rather than the rule. To address this issue, many studies estimate chromosome-specific somies from Illumina read alignments, often incorporating corrections for local changes in read-depth coverage and chromosome length [6,39]. Some authors have used an Expectation-Maximization (EM) approach, fitting a coverage model to read alignments for each isolate [40,41]. In such cases, manual inspection of alignment profiles is recommended when model overfitting is suspected, particularly in cases with high variance. For downstream analyses, software must allow specification of somy (often referred to as ploidy in certain programs) separately for each chromosome. As most programs do not accept fractional somy values, these are typically rounded to the nearest integer [6,13,17,39].

Another relevant feature of *Leishmania* genomes is the abundance of different forms of genetic amplifications, including extrachromosomal circular or linear episomes (Figure 1B) and tandem gene arrays (Figure 1C). All these forms of amplification lead to local changes in read-depth coverage due to the collapse of reads from repetitive regions, particularly when using short-read sequencing platforms. Identification of small variants such as SNPs and indels and the estimation of their functional impact are all strongly influenced by such local changes in coverage, especially when the amplified genetic element is an episome or a gene array potentially subjected to selective forces. These scenarios make it difficult to distinguish true sequence variants from the systematic errors introduced by sequencing techniques, a key step in the detection of variants by using bioinformatic tools. Furthermore, variants identified within amplified regions potentially reflect the evolution of such regions in specific strains or lineages, and may be inappropriate when inferring phylogenetic relationships or population structure. To cope with these issues, studies only considered variants with a normalized read-depth coverage within one or two standard deviations of the chromosome’s normalized median [13] or predefined ranges centered also in the normalized median [39,41], emphasizing that those median values should be estimated for each chromosome rather than a single value for the entire genome.

Overcoming the challenges posed by the unique genetic features of *Leishmania* genomes has enabled the further application of methods traditionally used in epidemiology and population genetics to studies focusing on *Leishmania* parasites. In addition to the usual characterization of parasite lineages using SNP-based phylogenetic trees and networks, principal component analysis (PCA) and model-based approaches, such as that implemented in the ADMIXTURE [42] software, have also been used to better understand genome diversity and population structure [12,39,41,43,44,45,46]. The role of the geographic distribution of parasites in shaping their evolutionary history has also been explored using the isolation-by-distance (IBD) analysis, typically performed with the Mantel test [39,41]. The extent of clonality, recombination, and hybridization among circulating strains have been characterized in virtually all studies by using the proportion and distribution of heterozygous sites, with loss of heterozygosity (LOH) and linkage disequilibrium (LD) decay typically used as indicators of mode of reproduction and gene flow among closely related lineages. LOH refers to genomic regions where heterozygous sites become homozygous, often through recombination or gene conversion. LD describes the non-random association of alleles at different loci, with extensive LD indicating clonality and its decay suggesting genetic exchange and recombination.

## 4. Characterizing the Genome Diversity of Parasites from the *Leishmania* (*Leishmania*) Subgenus

In the following sections, we review the main studies applying NGS strategies to characterize the genome diversity of *Leishmania* parasites belonging to the *L.* (*Leishmania*) and *L.* (*Viannia*) subgenera in both the Old and New World. For this review, we focused mainly on studies comprising multiple isolates rather than one or a few representative genomes, since this is a key requisite to properly characterize genome diversity and population structure. Studies considered in this review are summarized in Table 1 and Supplementary Table S1.

### 4.1. The L. donovani/infantum Species Complex

The first studies using NGS data to characterize the genome diversity of *Leishmania* focused on the species of the *L. donovani*/*infantum* complex in the Old World, with a particular emphasis on *L. donovani* populations from the Indian subcontinent [13,47]. These studies addressed the need for more resolutive molecular markers to characterize *L. donovani* circulating strains, which were often too similar at the genetic level to use traditional markers [48,49,50]. As outlined in an earlier section, the study conducted by Downing et al. [13] provided the first reference genome for *L. donovani* and characterized the genome-wide variability among 17 clinical isolates from the Indian state of Bihar and the Terai region of Nepal. The authors found a relatively low level of genetic differentiation among these isolates, comprising over 3500 SNPs, of which, only 17% were located within protein-coding genes. Despite the reportedly low genetic variability, a PCA and a phylogenetic network based on protein sequences revealed differences among the isolates that were not observed in previous studies using traditional markers.

In a subsequent study, Downing et al. [47] compared the suitability of genome-wide SNPs and a panel of conventional microsatellite markers to characterize the genetic diversity of 33 samples, comprising the previous 17 *L. donovani* clinical isolates, eight new isolates from Nepal and eight additional *L. donovani*/*L. infantum* strains from different regions of the world. Several criteria were used to select 130 SNPs from the total 3549 identified in the 2011 study, including those that were physically separated by at least 200 bp and were not located in regions with suspected biases in GC content or subjected to structural variations. The authors found that even this relatively reduced set of SNPs allowed the clustering of isolates from the Indian subcontinent into three well-supported clades that could not be demarcated by using only the microsatellite markers. However, microsatellites were still able to discriminate among isolates from the two species or those from distant geographic regions. In this context, results obtained from SNPs were roughly congruent with those derived from microsatellite profiling. SNP results also suggested the possible occurrence of additional populations, highlighting the need for extended sampling.

**Table 1:** Main studies using NGS data to characterize the genome diversity of circulating *Leishmania* parasites.

Study	Main Species *	Region *	Isolates	NGS Platform	Reference
Downing et al., 2011	*L. donovani*	Indian Subcontinent	17	Roche’s 454 Illumina	[13]
Downing et al., 2012	*L. donovani*	Indian Subcontinent	33	Illumina	[47]
Imamura et al., 2016	*L. donovani*	Indian Subcontinent	204	Illumina	[43]
Valdivia et al., 2017	*L. amazonesis* *L. infantum*	Southeastern Brazil	5	Illumina	[51]
S L Figueiredo et al., 2019	*L. braziliensis*	Northeastern Brazil	10	Illumina	[52]
Franssen et al., 2020	*L. donovani* *L. infatum*	Worldwide	151	Illumina	[41]
Patino et al., 2020	*L. braziliensis*	Bolivia, Brazil, and Colombia	21	Illumina	[53]
Patino et al., 2020	*L. panamensis*	Colombia and Panama	22	Illumina	[54]
Salloum et al., 2020	*L. tropica*	Asia, Africa and the Middle East	18	Illumina	[55]
Van den Broeck et al., 2020	*L.braziliensis* *L. peruviana*	Peruvian Andes and Amazon Basin	67	Illumina	[11]
Llanes et al., 2022	*L. panamensis*	Colombia and Panama	43	Illumina	[39]
Hadermann et al., 2023	*L. aethiopica*	Ethiopia	20	Illumina	[44]
Heeren et al., 2024	*L. braziliensis*	Amazonian and Atlantic Forests	257	Illumina	[45]
Talimi et al., 2024	*L. tropica*	Morocco	14	Illumina	[56]
Gonzalez-Garcia et al., 2025	*L. braziliensis*	Colombia	13	Illumina	[57]
Gonzalez-Garcia et al., 2025	*L. braziliensis* *L. guyanensis* *L. peruviana* *L. panamensis*	Central and South America	205	Illumina, ONT	[46]

* These columns mention the main species and geographical regions in which the study focused. This does not consider additional species or regions that were sampled with low isolate numbers in certain studies, mainly for comparative purposes.

These works were followed by a comprehensive study that examined 204 *L. donovani* clinical isolates from the Indian subcontinent collected from 2002 to 2012 [43], including 98 isolates from India, another 98 from Nepal, and eight from Bangladesh. In this study, the authors reported a core population of 191 isolates from the lowlands of the three countries. This core group was composed of parasites that seemed to be closely related among them but notably distinct from a separate ISC1 lineage, mainly composed of isolates from the Nepalese highlands. Not surprisingly, the authors found a relatively low genetic variation, with ~5000 variable sites in the ISC1 clade and only 2418 SNPs within the core population. However, SNP-based phylogenetic analysis, allele frequency-based statistics, PCA, and model-based clustering revealed a significant genetic structure within the core population, characterized by nine additional clades (ISC2-10) and several isolates that were not clustered into these main clades, some of which were proven to have a hybrid nature. Notably, the core population was characterized by a lack of LD decay that resulted in a lack of detectable recombination within clades ISC2-7. Furthermore, using Bayesian models, the authors were able to infer divergence dates for the entire core population (mid-19th century) and the more modern lineages ISC2, ISC4, ISC5, and ISC6 (1960s). The estimated divergence date for the latter lineages coincides with the time when spraying of the insecticide DDT (dichlorodiphenyltrichloroethane) was suspended, which was associated with the origin of leishmaniasis outbreaks in the Indian subcontinent.

The global genome diversity of *L. donovani* was ultimately characterized by Franssen et al. [41], in a study that also revealed important insights on the divergence and population structure of *L. infantum*. The study considered 151 isolates from the *L. donovani*/*infantum* complex, including 86 *L. donovani* isolates with a wide geographic span, mostly representing the global distribution of the species. Although several isolates from the previous studies were also included, the authors sequenced samples collected in previously unexplored regions from Asia, Africa, and the Middle East. The availability of such detailed information enabled, for the first time, the formal definition of five specific phylogeographic groups for the species, designated Ldon1-5. These phylogeographic groups were defined on the basis of a strong IBD signal in a phylogenetic reconstruction based on SNPs and roughly correspond to some of the lineages defined in previous studies, although this new study considered a notably higher number of ~400,000 polymorphic SNPs. This relatively high number of SNPs was likely because the study also included 65 isolates from *L. infantum*, which is a different species, although it belongs to the same species complex. Most of the *L. donovani* isolates exhibited remarkably low heterozygosity, likely driven by frequent aneuploidy turnover and selfing, yet a substantial subset of isolates displayed elevated heterozygosity that, when complemented with allele frequency analyses, suggested recent inter-lineage hybridization. Unlike *L. donovani*, however, most of the *L. infantum* isolates were clustered on a single phylogeographic group (Linf1) with relatively stable linkage patterns and low IBD signal despite their vast geographic origins. Findings from this study suggest a strikingly low genome diversity in *L. infantum* from both the Old and the New World, consistent with a recent clonal expansion and widespread dispersal, although a few diverse strains were indeed found. In this species, these divergent strains appear to have diversified by hybridization and may be associated with certain lineages close to the split between *L. donovani* and *L. infantum*, suggesting ancient admixture and complex ancestry in the Eastern Mediterranean.

A remarkable contribution of studies exploring the *L. donovani* genome diversity is the detailed characterization of genetic features associated with treatment failure and the emergence of resistance to antimonials. Trivalent antimonial (Sb^III^) compounds and their less toxic pentavalent (Sb^V^) counterparts were the first-line treatment for leishmaniasis worldwide for many years. In India, their use was discontinued in favor of alternative antileishmanial drug miltefosine due to widespread resistance in circulating *L. donovani* strains causing VL, particularly in the state of Bihar [58,59]. All the studies mentioned earlier have reported specific genetic markers of drug resistance, in particular *L. donovani* lineages. Among these, Downing et al. [13] first reported the amplification of two chromosomal segments, the H-locus and MAPK amplicons, both encoding genes previously found to be involved in virulence and resistance to antimonials. All isolates from the core population described by Imamura et al. [43], which corresponds to the Ldon1 phylogeographic group defined by Franssen et al. [41], were found to contain a varying number of copies of the H-locus and MAPK amplicons, whereas those from the ISC1 group (Ldon2) lacked these amplifications. These findings have led to the hypothesis that Ldon1 strains are preadapted to antimonials due to long-term exposure, while ISC1 strains remain globally susceptible. This was later confirmed by laboratory experiments in which the Sb^III^-resistant phenotype was induced in strains from these lineages [14]. In addition to these amplifications, mutations inactivating the AQP1 gene, encoding an aquaglyceroporin that mediates Sb^III^ uptake, were also found in antimony-resistant isolates from lineages ISC4 and ISC5 [43].

### 4.2. Other L. (Leishmania) Species Complexes

Although substantial progress has been made in using WGS data to characterize the genomic diversity of strains from the *L. donovani*/*infantum* complex, far less is known about other pathogenic *L.* (*Leishmania*) species in the Old World. Although most studies addressing genetic diversity in this region are still conducted using traditional markers, a few recent studies with geographically narrower spans have shown that the use of WGS data also poses the same great advantages. For instance, a study examining 20 *L. aethiopica* isolates from Ethiopia found a notably high genome diversity for this species, despite it being endemic only to Ethiopia and the highlands of Kenya [44]. The occurrence of near-identical genomes paired with extensive LOH in most isolates suggested a predominantly clonal or asexual mode of reproduction in *L. aethiopica*, although certain lineages showed evidence of LD decay, indicating possible genetic exchange by meiotic-like recombination. The authors reported two strains that were likely interspecific hybrids between *L. aethiopica* and *L. donovani* and *L. tropica*, respectively. In these two isolates, hybridization was not only evident but also possibly recent, due to observation of extensive genome-wide heterozygosity in these isolates, interrupted by relatively few homozygous patches.

At least two studies have also used WGS data to explore the genetic diversity of *L. tropica* [55,56], another particularly heterogeneous species of *Leishmania*, affecting several countries from Asia, Africa, and the Middle East. Salloum et al. [55] examined 18 *L. tropica* genomes from nine countries and reported broad consistency between findings obtained from microsatellite markers and genome-wide SNPs. In agreement with previous studies in *L. donovani*, genome-wide SNPs enabled the definition of more specific subpopulations, some of which did not entirely correlate with the geographical origin of parasite isolates. Talimi et al. [56] examined 14 *L. tropica* isolates from Morocco and found relatively low genome diversity among 12 of these isolates, suggesting a possibly specific SNP pattern distinguishing the Moroccan *L. tropica* strains from those circulating in other endemic areas. Larger datasets with a wider geographic span are crucial to better understand the genome diversity of these and other *Leishmania* species in the Old World.

Very little is also known about the genome diversity of species from the *L.* (*Leishmania*) subgenus present in the New World, which include *L. mexicana*, *L. amazonensis* and *L. infantum*. A 2017 study [51] sequenced three *L. infantum* isolates and two unexpected *L. amazonensis* isolates sampled from dogs in a canine VL focus in southeastern Brazil. In this study, SNP-based PCA enabled the proper separation of isolates from each species. Bayesian phylogenetic analysis suggested that the *L. amazonensis* isolates were closely related to a previous strain sampled in 1973 in northern Brazil, a region where *L. amazonensis* is considered endemic. In agreement with previous studies in the Old World, this study demonstrated the feasibility of NGS strategies to better characterize strains from *L.* (*Leishmania*) species circulating in the New World. However, although the authors were able to characterize parasites causing the canine VL focus, the number of sequenced isolates was too small to study genome diversity and population structure, even in that specific Brazilian region. Further studies are needed to better understand the complex scenario of two intertwining *Leishmania* subgenera in the New World, focusing not only on the underrepresented species but also on geographical regions that are still largely unexplored.

## 5. Genome Diversity of *Leishmania* (*Viannia*) Parasites

NGS has deepened our understanding of the genomic diversity of *Leishmania* species belonging to the *L*. (*Viannia)* subgenus, mainly affecting Central and South America. In the next sections, we review comparative and population-scale genomic analyses that have revealed a highly dynamic landscape of ecological divergence, structural plasticity, cryptic hybridization, and complex reproductive strategies in this subgenus, some of which challenge classical taxonomic and evolutionary models.

### 5.1. The L. braziliensis/peruviana Complex

Most of the *L.* (*Viannia*) studies have focused on species of the *L. braziliensis/peruviana* complex, due to their wide distribution and clinical importance across tropical regions of South America, including Brazil, Bolivia, Peru, Colombia and Venezuela. The first of these studies analyzed the genetic diversity of ten *L. braziliensis* isolates from the state of Pernambuco in northeastern Brazil, previously categorized into distinct zymodemes through multilocus enzyme electrophoresis (MLEE) [52]. Whole-genome SNP analysis revealed substantial genetic variation (~95,000 to ~132,000 SNPs for the different isolates), and phylogenetic clustering into three distinct groups corresponding to the ecological habitats of parasites. Forest isolates (zymodemes Z72/Z75) formed a unique clade separated from the other eight isolates circulating in urban environments, which in turn formed two additional but closely related clades. Notably, the lower heterozygosity found in the two forest isolates suggested genetic drift driven by isolation within the forest environment. This study did not find that aneuploidy was a source of variation between isolates as it was largely restricted to chromosome 31, with only one isolate showing additional chromosome-somy gains. Altogether, the findings of this study showed that NGS-derived markers were more appropriate to reveal intra-specific patterns of genetic variation in comparison to MLEE, highlighting substantial genetic diversity in *L. braziliensis* mostly associated with geographic restrictions and complex transmission cycles involving several vertebrate hosts and vectors.

A subsequent study [11] analyzed whole-genome sequences of 67 isolates of *L. braziliensis*, *L. peruviana*, and interspecific hybrids collected from 47 different localities across the Peruvian Andes and Amazon basin. Their phylogenomic reconstructions revealed the existence of three major groups: one composed of lowland *L. braziliensis* parasites and two groups composed of *L. peruviana* parasites inhabiting different biomes. Interestingly, no clear subpopulations were found in *L. braziliensis,* suggesting high gene flow within this lineage. In contrast, a strong genetic differentiation was found between *L. braziliensis* and *L. peruviana*, the latter being a derivative lineage of admixed Amazonian ancestors. Andean *L. peruviana* populations showed a reduced diversity and genome-wide fixed SNPs, suggestive of predominantly clonal propagation and ecological specialization to montane environments. Hybrid isolates found in sympatric zones displayed chromosomal mosaicism, biparental inheritance of minicircles, and uniparental inheritance of maxicircles, indicating past meiotic-like recombination between Andean and Amazonian *Leishmania* species. These findings provided direct evidence of genetic diversification associated with changes in forestation driven by climate change, which ultimately alters the composition of vertebrate reservoirs and vectors. Furthermore, this study evidenced interspecific gene flow within *L*. (*Viannia)* species, illustrating how ecological divergence and secondary contact zones can facilitate hybridization.

In a subsequent study, Patino et al. [53] performed genome-wide analysis of 21 *L. braziliensis* isolates, comparing 14 Brazilian isolates obtained from public databases with one Bolivian and six Colombian clinical isolates. Phylogenomic analysis of these isolates suggested the occurrence of four clades that evidenced high intraspecific variability. Extensive aneuploidy was found mainly in Brazilian genomes in comparison to Colombian and Bolivian isolates, and large tracts of loss of heterozygosity were identified mainly in clades from Colombia and Brazil. Clade-specific SNP signatures, somy differences, and CNV profiles highlighted significant structural plasticity within the species, potentially favoring parasite adaptation to different ecological niches. In agreement with Van den Broeck et al. [11], these authors suggested that the physical separation of ecosystems caused by the Andes mountain range can be an important source of intraspecific diversification.

Heeren et al. [45] presented a continent-wide, genome-scale analysis of *L. braziliensis* population diversity, using whole-genome sequencing data from 257 isolates collected across diverse Neotropical ecosystems. One of their most significant findings was the clear genetic differentiation between Amazonian and Atlantic Forest parasite populations. The Amazonian group showed high heterozygosity, low LD, and median inbreeding coefficients near zero, as would be expected for a population with frequent meiotic recombination. In contrast, Atlantic parasites exhibited pronounced clonality, negative median inbreeding coefficients, slow LD decay, and elevated loss of heterozygosity likely caused by gene conversion events, suggesting long-term clonal propagation and population bottlenecks. CNV patterns also reflected population-specific signatures, with most CNVs being rare and under purifying selection, except that Amazonian parasites exhibited a distinct profile characterized by specific amplifications in beta-tubulin and GP63 genes. Notably, differences in CNV burden did not entirely correlate with effective population size, suggesting that chromosomal plasticity and genome compartmentalization in *Leishmania* may buffer deleterious variation. The authors used demographic modeling tools to estimate divergence times and effective population size trajectories. The Amazonian and Atlantic populations likely diverged between 742 and 340 thousand years ago, shaped by Pleistocene climate shifts and biome fragmentation. This scenario is consistent with earlier hypotheses proposing the Amazon as a center of origin for *L. braziliensis* [11], followed by clonal expansion into degraded or fragmented environments such as the Atlantic Forest.

A recent study using NGS data in *L. braziliensis* aimed to understand treatment failure in clinical infections, revealed that natural resistance to antileishmanial drug amphotericin B (AmB) may involve distinct genomic mechanisms compared to those characterized in experimentally induced resistance models [57]. Thirteen *L. braziliensis* clinical isolates were obtained from CL patients unresponsive to antimony therapy. These isolates displayed variable in vitro susceptibility to AmB in both the promastigote and intracellular amastigote stages. Notably, two isolates exhibited significantly increased AmB tolerance in the amastigote stage. Whole genome sequencing revealed that tolerant isolates lacked canonical AmB resistance mutations, previously described in *L. donovani*, *L. infantum*, and *L. mexicana*. Instead, genomic comparisons identified novel SNPs and CNVs in genes associated with oxidative stress response and folate/biopterin metabolism. These findings suggested that natural AmB tolerance in *L. braziliensis* may be driven by mechanisms involving membrane remodeling and metabolic adaptation. Importantly, these results underscore the need to study clinical isolates directly, as their resistance profiles may not be adequately predicted by experimental models.

### 5.2. The L. panamensis/guyanensis Complex

A growing number of studies using NGS-derived data have focused on species of the *L. panamensis*/*guyanensis* complex, which are predominantly distributed across Central America and Northern South America. Initial studies analyzing clinical isolates of *L. panamensis*, the most common *Leishmania* species circulating in Panama and Colombia, mainly assessed genetic variation in the context of its potential association with virulence and antimony resistance [16,18]. However, the limited number of isolates in these studies precluded drawing robust conclusions regarding the population structure of *L. panamensis*. A subsequent study from Patino et al. [54], first attempted to explore the intraspecific genetic variability of circulating *L. panamensis* parasites from Panama and Colombia. Authors analyzed whole-genome data from 19 *L. panamensis* clinical isolates collected in Colombia and three publicly available genomes from Panama from a previous study [18]. SNP-based phylogenetic analysis identified three well-differentiated genetic clades that evidenced important levels of intraspecific diversity. In addition, the authors proposed that the Colombian lineages might have exhibited adaptation to human infection, inferred from their comparatively reduced SNP counts. However, the markedly smaller representation of Panamanian isolates in the dataset limited the extent to which those findings could be extrapolated to the species as a whole.

In order to better describe the genomic variability of *L. panamensis* across Panama and Colombia, Llanes et al. [39] analyzed the 19 previously published Colombian isolates jointly with 24 *L. panamensis* isolates from several Panama provinces. The phylogenetic reconstructions in this study confirmed the existence of three well-supported groups highly correlated with geographical location, designated Lpan1-3. Lpan1 grouped most of the Panamanian isolates, while Lpan2 and Lpan3 grouped most of the Colombian isolates. Furthermore, admixture analysis suggested the presence of additional groups in Panama whose occurrence agrees with the IBD relationships estimated for all isolates. Additionally, strong evidence was found for the mixed origin of several isolates within groups Lpan1 and Lpan3, although the proportion of heterozygous sites between the two groups varied considerably, probably due to varying frequencies of inbreeding across subpopulations. The evaluation of several population genetics parameters showed low genetic flow between the main groups, inbreeding coefficients greater than zero, and values of LD suggesting a non-panmictic population structure but lower than expected for clonal populations. Altogether, these results supported the existence of a mixed mode of reproduction involving clonality and sporadic meiotic-like recombination events occurring mainly within subpopulations of closely related strains.

### 5.3. Integrating the Main L. (Viannia) Species Complexes

In a more recent comprehensive study, González-García et al. [46] conducted an expanded genomic characterization of *Leishmania* (*Viannia*) species, with a particular emphasis on clinical isolates circulating in Colombia. The authors integrated whole-genome sequencing data from 205 samples that included 65 newly sequenced Colombian isolates, comprising previously uncharacterized *L. braziliensis* isolates from Eastern and Central Colombia and *L. panamensis* isolates from Northwestern Colombia. Phylogenomic analyses based on ~500,000 SNPs identified cryptic population subdivisions within Colombian *L. braziliensis* and *L. panamensis* isolates correlating with distinct ecological regions, which agreed with previous studies [11,39,45]. Notably, *L. braziliensis* populations appeared to exhibit greater genetic variation compared to *L. panamensis* and *L. guyanensis.* The two clusters observed in Colombian *L. braziliensis* corresponded to their geographic origin, separating Andean parasites from those circulating in the Orinoco and Amazon regions. Group delimitations in *L. panamensis* were consistent with Llanes et al. [39], but a new Lpan4 group was identified, containing isolates from the Colombian Eastern Andean mountain range. The presence of admixed *L. panamensis*/*L. braziliensis* and *L. guyanensis*/*L. braziliensis* isolates underscored the potential role of exchange of genetic material in shaping *Leishmania* genomic architecture and population structure. Furthermore, this study shows the scalability of genome-wide approaches when characterizing not just individual species complexes but entire subgenera, possibly incorporating currently unexplored geographic regions.

## 6. Towards an Enhanced Characterization of Population Structure and Mode of Reproduction

The shift from classical genotyping methods to NGS-derived methods has greatly enhanced resolution for detecting fine-scale structure, hybridization, and adaptive variation in *Leishmania*. This enhanced resolution is key to understand the potential outcomes of hybridization, including the mixing of genetic material from distinct populations, termed admixture, or the more stable incorporation of genetic material from one population into another through repeated backcrossing, termed introgression. Historically, methods such as MLEE, MLMT, and MLST provided valuable but coarse snapshots of diversity, identifying major lineages, detecting strong clonality, and providing phylogeographic insights [60,61]. These approaches suffer from limited genomic coverage, homoplasy, and low resolution, often failing to capture fine-scale population structure variation or recent recombination events. Genome-wide NGS approaches, by contrast, provide hundreds of thousands of markers and enable simultaneous analysis of SNPs, CNVs, and somy variation, allowing direct detection of genomic signatures related to adaptation and other processes. High-density SNP datasets have enabled fine-scale estimation of LD decay, effective population size, and recombination rates, while also detecting subtle admixture, hybridization tracts, structural variants, and somy changes [4,6,11,39,41,43,45,61]. These genomic resources have revealed patterns invisible to classical markers, such as cryptic hybrids, population-specific copy number variation, and recombination hotspots [11,39,41,45,46]. Nonetheless, NGS introduces its challenges, including reference bias in cross-subgenera mapping, paralog misalignment in multicopy families, the need for somy-aware SNP calling to avoid ploidy-driven artifacts, and challenges to differentiate true recombination from gene conversion [39,41,45,46,62].

*Leishmania* population structure has been framed within a spectrum of interpretations ranging from strict clonality to frequent meiotic recombination, often within the same species. Evidence from different studies using field and laboratory strains has confirmed that *Leishmania*’s reproductive repertoire is more diverse than just strictly clonal (see Ferreira [63] for an in-depth review). Both inter- and intraspecies natural hybrids across *L.* (*Leishmania*) and *L*. (*Viannia*) subgenera have been described [11,39,41,44,64], with potential impacts in parasite ecology ranging from enhanced virulence to expansion of parasite populations onto new geographical ranges through adaptation to diverse host-vector systems [63]. Additionally, whole-genome level SNP analyses have revealed sharply contrasting evolutionary patterns between major *Leishmania* lineages. While some populations exhibit high heterozygosity coupled with rapid LD decay and near-zero inbreeding coefficients, indicative of recurrent genetic exchange by random mating [11,41], other populations show extended LOH and slow LD decay, which are indicators of long-term clonal propagation and historical bottlenecks coupled with varying frequencies of inbreeding events within populations [11,39,41,45]. Such patterns underscore that *Leishmania* evolution cannot be reduced to a binary of clonality versus sexuality and, rather, a predominant clonal evolution (PCE) framework appears to apply across many taxa [61]. This paradigm holds that while *Leishmania* retains the ability to undergo genetic recombination, its evolutionary trajectory is dominated by clonal propagation, producing long-lasting LD and stable multilocus genotypes that persist across broad spatial and temporal scales. Occasional recombination events, whether sexual or parasexual, do occur, but are typically rare and insufficient to erase the strong population subdivision characteristic of PCE systems. In the context of this paradigm, the stable evolutionary units whose discreteness have been somewhat blurred by occasional recombination are called near-clades. These near-clades form the basic units of *Leishmania* population structure, each with its own evolutionary history and often distinct ecological and epidemiological traits.

Genetic exchanges in *Leishmania* can occur through classical meiotic-like recombination, but also via non-Mendelian processes such as polyploid hybrid formation and horizontal gene transfer. Meiotic-like recombination, which leads to the emergence of Mendelian hybrids (including selfing and backcrossing), has been shown to occur through controlled crossing experiments of laboratory strains during propagation in sandfly vectors [65,66,67,68]. Louradour et al. [69] conducted sand fly co-infection experiments using *L. tropica* as a model, and showed that hybrid formation is not confined to a specific sand fly species but occurs across multiple permissive vector species. Moreover, these authors demonstrated that genetic exchange can be induced in vitro between axenic promastigotes, with hybridization frequencies greatly increased under stress conditions (e.g., DNA damage, heat shock, nutrient deprivation). These hybrids inherit nuclear DNA biparentally but mitochondrial DNA uniparentally, often display polyploidy, and maintain viability and transmissibility in sand flies. This evidence suggests that parasexual processes, such as cell fusion followed by chromosome loss, may complement meiotic-like mechanisms in generating genomic diversity in *Leishmania*.

Adding further complexity, non-meiotic transfer of genetic material has been described in *Leishmania* [70]. One of the mechanisms allowing this transfer is called vesiduction and it is defined as the extracellular vesicle-mediated transfer of large DNA fragments between parasites, enabling horizontal gene transfer of genetic elements without forming full genomic hybrids. Douanne et al. [70] proposed that vesiduction may potentially allow for the rapid acquisition of adaptive traits across lineages and even species boundaries, such as drug resistance genes or host-specificity determinants. Under controlled laboratory conditions, the authors showed in wild-type and drug-resistant strains that fragments from almost the entire genome of each strain were packed into extracellular vesicles. Nonetheless, extracellular vesicles from drug-resistant *Leishmania* were enriched with circular DNA amplicons containing resistance genes. Resistance genes were shown to be transferred via transwell co-culture, direct exposure to purified extracellular vesicles, and nucleofection, leading to changed gene expression, increased drug resistance, and improved growth/oxidative-stress tolerance. Interestingly, extracellular vesicles conferred strong protection to DNA fragments, even after DNAse treatment, and accommodated DNA fragments up to 16 kb. Because vesiduction can occur between strains of different species or subgenera, it offers a plausible explanation for certain mosaic genomic patterns that are not easily reconciled with meiotic hybridization alone. Together, these findings suggest that *Leishmania*’s genome evolution is shaped by a combination of predominantly clonal propagation, rare meiotic recombination, parasexual polyploid cycles, and horizontal gene transfer events.

Interpretations of reproductive mode are tightly coupled to the metrics and assumptions underlying population-genetic analyses. Rapid LD decay, high heterozygosity, and minimal inbreeding are taken as signatures of frequent meiotic recombination and gene flow, whereas slow LD decay, reduced heterozygosity, and deep clonal clusters are interpreted as evidence of PCE. Conversely, even low levels of sexual recombination can disrupt clonal structure, making it challenging to distinguish between predominantly clonal and panmictic populations without large, well-designed datasets [62]. Many of the distinctive features of *Leishmania* genomes discussed earlier violate the assumptions of standard diploid models, distorting allele frequency distributions, heterozygosity estimates, and linkage patterns [4,6,11,39,41,43,45,61]. Mixed reproductive systems further confound interpretation because methods assuming Hardy–Weinberg equilibrium, such as admixture analysis, tend to misestimate migration, effective population size, and divergence times when clonality predominates [45,71]. Hybridization and co-infections introduce additional complexity as structural genome plasticity can shape post-hybridization evolution through extensive aneuploidy variation, which ultimately affects the retention of heterozygosity [62,69]. Furthermore, allele mixtures can mimic either admixture or contamination unless haplotype-aware and local-introgression methods are applied [11,46].

Best-practice recommendations for improving population genetic inference in predominantly clonal or partially clonal pathogens emphasize the importance of proper method selection, somy modeling, and validation across complementary approaches. Model-based clustering approaches are inherently problematic for clonal or partially clonal populations and should be validated with resampling and nonparametric methods, particularly under uneven sampling [62,71,72]. Unsupervised methods robust to some model violations, such as k-means clustering, PCA, and discriminant analysis of principal components (DAPC), should be used to cross-validate population assignments from model-based clustering methods [62,72]. All previous methods should be combined with clonality-aware metrics that include the ratio between the number of multilocus genotypes and the number of individuals in a population (G/N ratio), and multilocus linkage indices such as the standardized index of association [72]. Over-representation of identical genotypes is usually avoided by applying clone-censoring prior to diversity and LD analyses [62]. This method implies the reduction in a dataset to a single representative observation of each multilocus genotype, to avoid biases in diversity and linkage disequilibrium estimates.

Hybridization and introgression require haplotype-based approaches or comparison of allele frequencies between populations through local D-statistics to identify heterozygosity tracts [73,74]. Ideally, CNVs should be corroborated with long-read assemblies or orthogonal assays rather than inferred solely from short-read depth [46]. Finally, a proper sampling of the target population remains critical, considering geographic coverage, ecological representation, and inclusion of vector/reservoir isolates, since poor population representation can obscure or distort inferred structure [62]. Integrating these methodological safeguards into NGS-era data analyses will yield more accurate and biologically meaningful inference of *Leishmania* population structure and reproduction modes.

## 7. Future Directions

Over the past two decades, NGS strategies have been key in characterizing the genome diversity of circulating *Leishmania* strains. WGS studies encompassing tens to hundreds of isolates have provided unprecedented insights into the biology, epidemiology, population structure, and evolutionary dynamics of a parasite with highly distinctive genomic features. In the Old World, WGS efforts have concentrated mainly on the *L. donovani/infantum* complex, while in the New World they have focused largely on species of the *L. (Viannia)* subgenus. Compared with more traditional markers, genome-wide SNPs have revealed much finer-scale population structure, often associated with geographic origin, hybridization, and drug resistance profiles. These findings have transformed the conceptual framework of *Leishmania* population structure from a model of strict clonality to one recognizing a dynamic interplay of clonal propagation with sporadic sexual and parasexual recombination, whose frequency and impact vary by species and ecological context. Future progress will rely on integrative, clonality-aware approaches considering that *Leishmania* evolution is shaped by microevolutionary trajectories heavily influenced not only by a highly dynamic genome, but also by constant bottleneck events due to complex life cycles.

Additionally, emerging technologies offer powerful tools to overcome current limitations in the study of *Leishmania* population structure and evolutionary dynamics. Single-cell genomics can aid in uncovering cellular heterogeneity and rare subpopulations, shedding light on developmental plasticity and recombination events. Long-read sequencing could help resolve structural variants and repetitive regions, enhancing comparative analyses across isolates. Pangenome approaches, which move beyond single-reference models to capture core and accessory genomic content, may ease results comparison between different inter-species studies. Finally, integrating multi-omics datasets can connect genetic diversity to functional outcomes, aiding in clarifying the interplay between genome architecture, gene expression, and population structure across endemic regions.

A notable research gap is evident for a number of species that have not been studied enough but cause significant morbidity in humans. Species from the well-known *L*. (*Leishmania*) subgenus, such as *L*. *amazonensis*, and *L*. *mexicana*, are examples of those requiring much more sampling efforts and extensive genomic characterization. Similarly, species from the recently created subgenus *L*. (*Mundinia*), such as *L*. *martiniquensis* and *L*. *orientalis*, may also cause disease in humans. Although recent studies have described the genomes of some of these species based on reference specimens, not enough sampling has been performed to allow for a thorough characterization of their genome diversity across populations and/or geographical areas, as well as to gain knowledge about clinically important traits such as drug resistance and range of vectors and reservoirs. New studies should focus on a broader and more comprehensive geographic sampling, while giving greater attention to unexplored species and underrepresented regions that may hold key insights into parasite diversity and evolution.

## Data Availability

No new data were created.

## Supplementary Materials

The following supporting information can be downloaded at: https://www.mdpi.com/article/10.3390/life15101590/s1, Table S1: Main findings of studies included in this review.

## Abbreviations

The following abbreviations are used in this manuscript:

AmB	Amphotericin B
CL	Cutaneous leishmaniasis
CNV	Copy number variation
DAPC	Discriminant analysis of principal components
DDT	Dichlorodiphenyltrichloroethane
EM	Expectation-Maximization
IBD	Isolation by distance
LD	Linkage disequilibrium
LOH	Loss of heterozygosity
MAPK	Mitogen-activated protein kinase
MLEE	Multilocus enzyme electrophoresis
MLMT	Multilocus microsatellite typing
MLST	Multilocus sequence typing
NGS	Next-generation sequencing
MCL	Mucocutaneous leishmaniasis
PCE	Predominant clonal evolution
ONT	Oxford Nanopore Technologies
PCA	Principal component analysis
SNP	Single-nucleotide polymorphism
VL	Visceral leishmaniasis
