# Supplementary material for: Unraveling the Genome Diversity of Leishmania Parasites Using Next-Generation DNA Sequencing Strategies
Source: Life (Basel). 2025 Oct 11;15(10):1590. doi: 10.3390/life15101590 (PMC12565333; doi:10.3390/life15101590)
Supplement: Supplementary file 1 [file life-15-01590-s001.zip › life-3850096-supplementary.pdf]

Table S1

Main findings of studies included in this review.

Study	Reference	Type of study	Main findings
Downing et al., 2011	[13]	Whole-genome sequencing of 17 *L. donovani* clinical isolates from the Indian subcontinent	Revealed relatively low level of genetic differentiation (3,500 SNPs). PCA and phylogenetic analyses revealed differences among isolates that were not observed by using more traditional markers such as MLEE.
Downing et al., 2012	[47]	Whole-genome SNP analysis of 33 isolates from the *L. donovani*/*L. infantum* complex mainly from the Indian subcontinent	Confirmed the higher resolution of genome-wide SNPs over MLEE in the demarcation of specific clades among circulating *L. donovani* strains
Imamura et al., 2016	[43]	Whole-genome SNP analysis of 204 *L. donovani* clinical isolates from the Indian subcontinent collected from 2002 to 2012	An extension of the previous to studies from Downing et al., this study defined a core population and up to 10 well defined clades (ISC1-10), some of which seem to have originated due to geographic isolation. The origin of some of these lineages was traced to the time were the use of DDT as insecticide was suspended in the 1960s.
Valdivia et al., 2017	[51]	Whole-genome sequencing of 5 isolates (*L. infantum* and *L. amazonensis*) from dogs from southeastern Brazil	SNP analysis allowed for the proper sepatation of isolates from each species, with the first report of L. amazonensis isolated from cases of canine VL, in a region where this species is not considered endemic.
S L Figueiredo et al., 2019	[52]	Whole-genome SNP analysis of 10 *L. braziliensis* isolates from Pernambuco, Brazil	Identified ~95,000–132,000 SNPs; three phylogenetic groups corresponding to ecological habitats (forest vs. urban). The lower heterozygosity in forest isolates suggested genetic drift. NGS markers were superior to MLEE for revealing intra-specific diversity.
Franssen et al., 2020	[41]	Whole-genome SNP analysis of 151 *L. donovani*/*infantum* isolates with a worldwide distribution	Confirmed the occurrence of well-supported *L. donovani* phylogeogaphic goups with a strong IBD signal, named with the Ldon prefix. Unlike *L. donovani*, *L. infantum* isolates were clustered into a single phylogeographic

			group despite coming from different regions of the world, suggesting a probably lower genetic divergence.
Patino et al., 2020	[53]	Whole-genome sequencing of 21 *L. braziliensis* isolates (Brazil, Bolivia, Colombia)	Identified four phylogenomic clades with high intraspecific variability. Brazilian isolates showed extensive aneuploidy, and Colombian and Brazilian isolates had large LOH regions. Clade-specific SNPs and CNVs suggested structural plasticity enabling adaptation. The authors suggested that the Andes mountain range acts as a barrier promoting diversification.
Patino et al., 2020	[54]	Genome-wide analysis of 19 *L. panamensis* isolates from Colombia and 3 isolates from Panama	Identified three distinct clades. Colombian lineages showed reduced SNP counts, suggesting adaptation to human infection. The limited Panamanian representation restricted broader conclusions.
Salloum et al., 2020	[55]	Whole-genome sequencing of 18 *L. tropica* genomes from 9 countries	Identified several subpopulations, some of which did not entirely correlate with geographical origin. Corroborated previous reports of the highly heterogeneous nature of this species at the genetic level.
Van den Broeck et al., 2020	[11]	Whole-genome sequencing of 67 isolates (*L. braziliensis*, *L. peruviana*, and hybrids) from Peru	Revealed three major groups (lowland *L. braziliensis* and two *L. peruviana* lineages). High gene flow was found within L. braziliensis but a strong differentiation from *L. peruviana*. *L. peruviana* showed reduced diversity and clonal propagation while hybrids displayed chromosomal mosaicism and evidence of meiotic-like recombination. Results linked diversification to ecological changes driven by climate change.
Llanes et al., 2022	[39]	Phylogenomic and population genetics of 43 *L. panamensis*	Confirmed three main groups (Lpan1–3) associated with geographical distribution. Admixture analyses

		isolates (Colombia and Panama)	suggested the existence of additional groups and some mixed origin isolates were found within Lpan1 and Lpan3 groups. Population structure analysis showed limited gene flow, inbreeding, and sporadic meiotic-like recombination alongside clonality.
Hadermann et al., 2023	[44]	Whole-genome analysis of 20 *L. aethiopica* from Ethiopia	Revealed a high genome diversity in circulating strains, despite the relatively small endemic area for this species, with at least two possible hybrids, one with *L. donovani* and another one with *L, tropica*.
Heeren et al., 2024	[45]	Continent-wide population genomics of 257 *L. braziliensis* isolates across Neotropical ecosystems	Clear differentiation between Amazonian (high recombination, heterozygosity) and Atlantic Forest (clonality, LOH) populations. CNV patterns were largely under purifying selection. The divergence between Amazonian and Atlantic populations dated to 742–340 kya and was linked to Pleistocene biome shifts.
Talimi et al., 2024	[56]	Whole-genome sequencing of 14 L. tropica isolates from Morocco	Identified a SNP pattern possibly distinguish *L. tropica* isolates circulating in Morocco.
Gonzalez-Garcia et al., 2025	[57]	Whole-genome sequencing of 13 clinical *L. braziliensis* isolates from patients failing antimony therapy	Variable amphotericin B (AmB) susceptibility. Resistant isolates lacked canonical resistance mutations seen in other species and novel SNPs/CNVs implicated in oxidative stress and folate/biopterin metabolism were described. The findings highlighted the existence of unique natural resistance mechanisms and the importance of studying clinical isolates.
Gonzalez-Garcia et al., 2025	[46]	Expanded genomic characterization of 205 *Leishmania* (*Viannia*) isolates (mainly Colombian	Identified cryptic subdivisions correlated with ecological regions and *L. braziliensis* showed greater variation than *L. panamensis*/*L. guyanensis*. Colombian *L. braziliensis* clustered into

	L. braziliensis and *L. panamensis*)	Andean vs. Amazon/Orinoco lineages. The study confirmed Lpan1–3 groups, described a new Lpan4 group, and detected admixed isolates suggesting recombination and introgression shaping diversity.
